# Clinical Efficacy and Safety of Total Glucosides of Paeony for Primary Sjögren's Syndrome: A Systematic Review

**DOI:** 10.1155/2017/3242301

**Published:** 2017-05-31

**Authors:** Liang Jin, Chengyin Li, Yanping Li, Bin Wu

**Affiliations:** ^1^Department of Rheumatology, Chongqing Hospital of Traditional Chinese Medicine, Chongqing 400021, China; ^2^Graduate School, Hunan University of Traditional Chinese Medicine, Changsha 410007, China; ^3^Chongqing Engineering Technology Research Center of Traditional Chinese Medicine Characteristics on Diagnosis and Treatment, Chongqing 400021, China

## Abstract

**Objective:**

To evaluate the clinical efficacy and safety of total glucosides of paeony (TGP) for primary Sjögren's syndrome (pSS).

**Methods:**

Eight electronic databases were searched from their inception to July 2016. Clinical randomized controlled trials (RCTs) were included. The study quality was evaluated according to the standard suggested in the Cochrane Handbook. RevMan 5.1 was used for statistical analysis.

**Results:**

Seven RCTs involving 443 patients were included. The results showed that TGP combined with an immunosuppressant (IS) showed greater efficacy for improving the saliva flow test of pSS compared to immunosuppressant alone (WMD −6.88, 95% CI −9.02 to −4.74, and *P* < 0.00001). And the same trend favouring TGP-IS dual combination was found in Schirmer test (WMD 1.63, 95% CI 0.26 to 3.01, and *P* = 0.02), ESR (WMD 7.33, 95% CI −10.08 to −4.59, and *P* < 0.00001), CRP (WMD −6.00, 95% CI −7.17 to −4.83, and *P* < 0.00001), IgM (WMD = −0.42, 95% CI −0.70 to 0.13, and *P* = 0.004), and IgG (WMD −3.22, 95% CI −4.32 to −2.12, and *P* < 0.00001) analysis. However, TGP did not affect IgA (WMD 0.53, 95% CI −1.34 to −0.29, and *P* = 0.20). The adverse events manifested no significant differences between the two groups.

**Conclusions:**

The TGP-IS combination is superior to IS alone in the treatment of pSS. However, due to the low quality of included studies, high-quality RCTs are needed to confirm the beneficial effects of TGP.

## 1. Introduction

Primary Sjögren's syndrome (pSS) is a chronic systemic autoimmune disease characterized by lymphocytic infiltration of the exocrine glands [1]. Recent epidemiological studies reported that pSS is the second most common rheumatic disease in China with a prevalence of about 0.77% among the general population [2]. At least one-third of patients may develop extraglandular manifestations such as renal tubular acidosis and interstitial lung disease, which could lead to lethal effects [3]. Consequently, many patients experience poor quality of life, and many experts are dedicated to finding new drugs [4, 5].

Although the pathogenesis of pSS is not yet entirely clear, it is widely believed that abnormal immunity plays the most important role in the pathogenesis of pSS [6]. Regarding therapeutic options in the clinic for patients with pSS, treatment tends to focus on symptomatic relief, supportive measures, and prevention of local complications [7]. Of the currently available pharmacological interventions, disease-modifying antirheumatic drugs (DMARDs) are the main therapies, among which cyclosporine and corticosteroids are often selected to treat refractory pSS. However, there is a risk of adverse events associated with DMARD treatment. Besides, evidence for the effectiveness of DMARD therapies for pSS is limited [8]. Therefore, safer and more effective drugs need to be found.

*Paeonia lactiflora* Pall (Bai-Shao in Chinese) is a Chinese herbal medicine with proven antioxidative and neuroprotective effects, which is widely used in oriental countries [9]. Total glucosides of paeony (TGP) are generally considered as the major active compound found in* P. lactiflora* Pall. To date, several researchers have summarized the pharmacological effects of TGP on autoimmune diseases such as pSS [10], rheumatoid arthritis [11], and systemic lupus erythematosus [12]. In China, Zhang et al. [13] reported that TGP is effective for pSS and causes few adverse reactions in patients. In addition, some studies found that TGP can also reduce the liver toxicity induced by immunosuppressive drugs [14–16]. Therefore, in the clinic, TGP is often combined with other immunosuppressive agents. In general, the pharmacological effects of TGP are related to its anti-inflammatory and immunoregulatory effects. It influences cell immunity, humoral immunity, and inflammation processes through several approaches and targets [17]. As a result, it has reliable curative effects on such autoimmune diseases. Consequently, TGP may represent a promising therapeutic option for the treatment of pSS. Given the lack of systematic reviews of the use of TGP for pSS, we carried out this study to assess the effectiveness and safety of TGP in the treatment of pSS.

## 2. Methods

### 2.1. Search Strategy

The following databases were searched from their inception to July 2016: PubMed, Cochrane Central Register of Controlled Trials, Cochrane Database of Systematic Reviews, ISI Web of Knowledge, Chinese Biomedical Database, Chinese National Knowledge Infrastructure (CNKI), WanFang Database, and the Chongqing VIP Information Database (VIP). References in the reports identified were also searched. For the English databases, subject headings and text-word searches were used, and the search details included “primary Sjögren's Syndrome”, “pSS”, “total glucosides of paeony”, “TGP”, “randomized controlled trial”, and “RCT” and their synonyms. For the Chinese-language searches in the CNKI, VIP, and WanFang Databases, the same search strategy and search terms were used.

### 2.2. Selection Criteria

*Type of Study*. Full-text randomized controlled trials (RCTs) investigating the use of TGP for pSS in China and other countries were included, regardless of blinding, or types and languages of publication.* Subjects*. Study population included patients with pSS, regardless of sex, age, or ethnicity. A diagnosis of pSS was in line with the international classification of pSS in 2002 [18].* Interventions*. Patients in the experimental group received oral TGP in combination with immunosuppressant (IS) treatment. In the control group, patients were treated with IS alone.

### 2.3. Outcome Measures

Outcome measures included the percentage of patients with improved clinical symptoms, experimental results (Schirmer test, saliva flow test), inflammatory markers such as C-reactive protein (CRP) and erythrocyte sedimentation rate (ESR), immunoglobulin (IgG, IgM, and IgA), and adverse reactions.

### 2.4. Data Extraction and Study Quality Assessment

Two reviewers (L. Jin and C. Y. Li) screened all titles and abstracts of the studies independently. Full texts of potentially included studies were retrieved for further identification according to the eligibility criteria. Data were extracted from all included studies using a standardized extraction form especially created for this meta-analysis. The form contained information on the participants, the methodological aspects of the study, interventions, and measured outcomes. Disagreements were resolved by discussion and by consultation with other authors, and a judgment was made based on consensus. Finally, these forms were merged into a single extraction form.

The study quality was evaluated as suggested in the Cochrane Handbook in terms of randomization [19], allocation concealment, blinding, completeness of data, and selective reporting, which were employed to evaluate the quality of the RCTs. “Reported”; “unclear”; or “not reported” were used to determine the standards mentioned above.

### 2.5. Statistical Analysis

Review Manager 5.1 downloaded from the website of Cochrane collaboration (http://www.cochranelibrary.com/) was used for data analysis. Available data of sufficient quality and similarity were used for a meta-analysis. Dichotomous data were expressed as relative risks (OR). Continuous data were expressed as weighted mean differences (WMD). A fixed-effect or random-effect model was used according to the heterogeneity. Heterogeneity was tested using the *Z* score and the Chi-square statistics with significance set at *P* < 0.1. The fixed-effect model was used to combine dichotomous data if the data were homogeneous. Conversely, the random-effect model was used otherwise.

## 3. Results

### 3.1. Results of Literature Search and Characteristics of Included Studies

**Figure 1:**
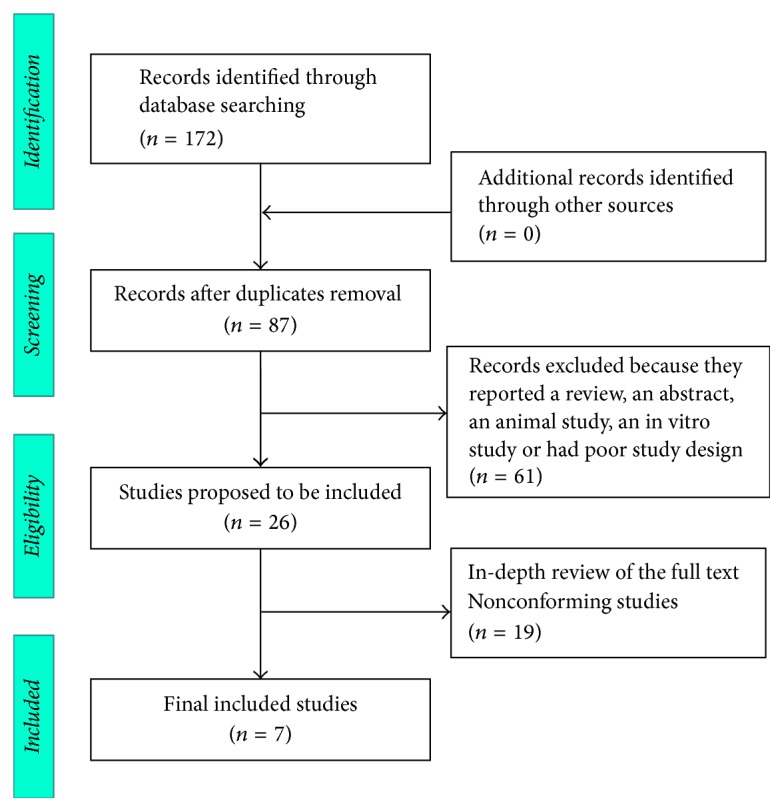
Flow chart of inclusion and exclusion criteria and study selection.

A total of 172 relevant publications were retrieved by screening titles and reviewing full texts. According to the aforementioned screening criteria and double assessment by two reviewers, seven randomized controlled trials met the inclusion criteria [20–26], which included 443 cases (230 cases in the experimental group and 213 cases in the control group). The literature screening process is illustrated in Figure 1. The characteristics of the selected reports are shown in Table 1.

### 3.2. Quality Assessment of Included Studies

**Figure 2:**
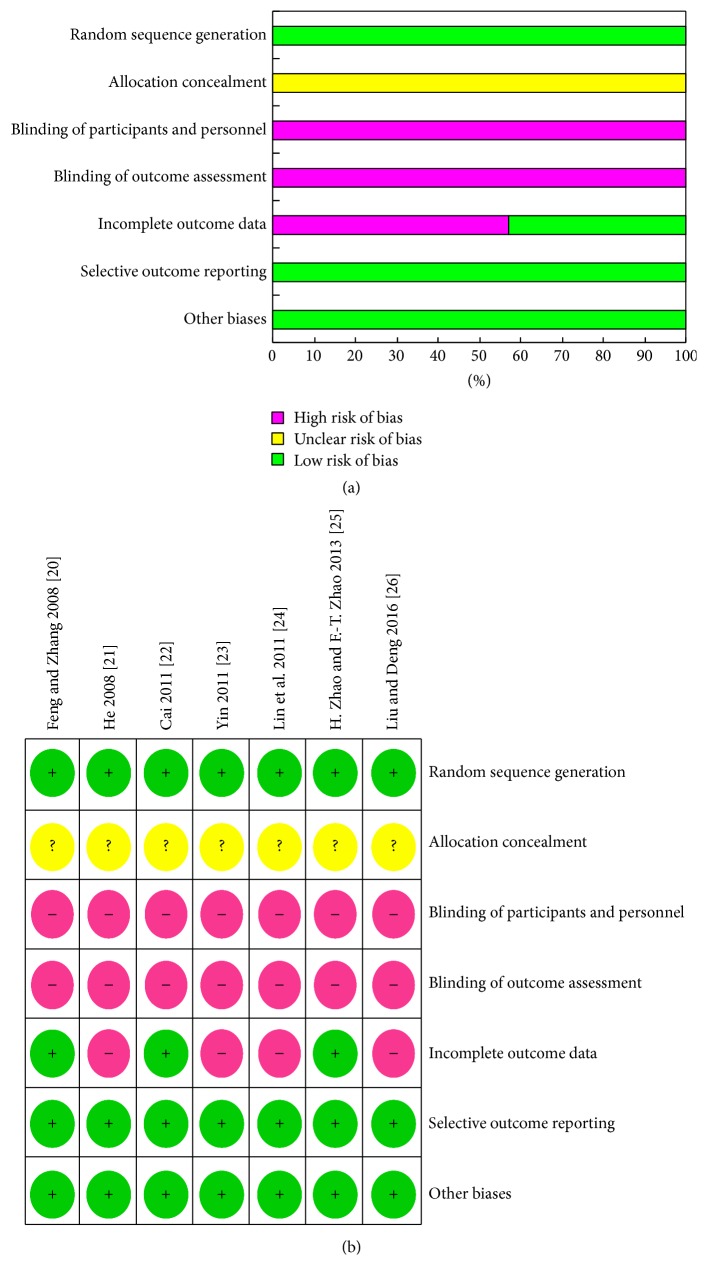
Risk of bias assessment results. (a) Risk of bias graph: review of authors' judgments about each risk of bias item presented as percentages across all included studies. (b) Risk of bias summary: review of authors' judgments about each risk of bias item for each included study.* Note*. “?”: unclear, “+”: reported, and “–”: not reported.

Seven RCTs were evaluated [20–26]: overall qualities were basically acceptable, and the baseline characteristics of all patients were reported. All RCTs stated they were “random,” but only one reported the details of the random sequence generation [26]. No RCT reported adequate allocation concealment. Two of the included articles reported loss of follow-up or drop-out [20, 25]. Blinding was not mentioned in all trials. Since all RCTs had a high or moderate risk of bias, we have learned that six studies have been grouped with random numbers by contacting the authors of the other six articles. Therefore, the included studies basically met the quality requirements of meta-analysis, and the result is shown in Figure 2.

### 3.3. Results of Efficacy Evaluation

**Figure 3:**
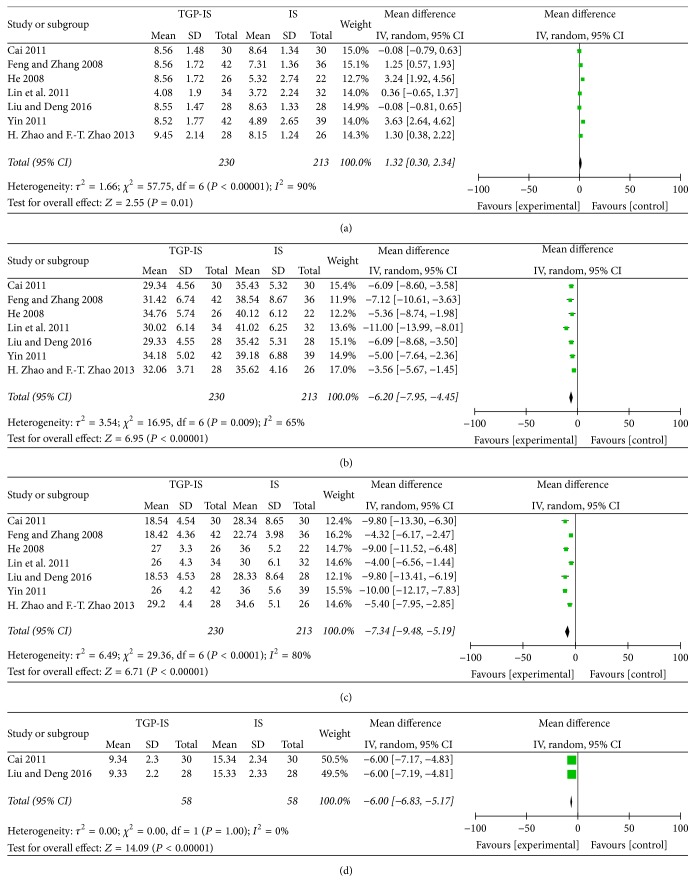
Forest plot of studies comparing TGP-IS group and the IS group, examining the effect on primary Sjögren's syndrome (including Schirmer test, saliva flow test, ESR, and CRP). (a) Schirmer test. (b) Saliva flow test. (c) ESR. (d) CRP.* Note*. TGP-IS group: the group treated with TGP in combination with an immunosuppressant. IS group: the group which received an immunosuppressant alone.

All studies [20–26] reported the effective rate of the treatment. However, it was not a standard outcome measure, while it was markedly affected by bias. For this reason, we gave up the effective rate statistical analysis. All studies [20–26] compared the changes in the results of the Schirmer test and saliva flow test. With significant heterogeneity, the random-effect model was used to evaluate treatment efficacy. As shown in Figures 3(a) and 3(b), the results suggest that patients treated with the TGP-IS combination showed better functional outcomes than those receiving IS alone, as evidenced by improvements in Schirmer test and saliva flow test (WMD 1.32, 95% CI 0.3 to 2.34, *Z* 2.55, and  *P* = 0.01; WMD −6.2, 95% CI −7.95 to −4.45, and *P* < 0.00001, resp.).

We analyzed the reductions of inflammatory indices reported in the RCTs. All studies [20–26] compared the changes of ESR between the TGP-IS group and the IS group. Heterogeneity was found among these studies (*I*^2^ = 80%, *P* < 0.00001), so the random-effect model was used. The result indicated that ESR was more significantly reduced in the TGP-IS group than in the IS group (WMD −7.34, 95% CI −9.48 to −5.19, *Z* 6.71, and *P* < 0.00001, Figure 3(c)). Only two studies [22, 26] compared the changes in CRP. Meta-analysis showed that TGP-IS therapy was more effective than IS single-agent therapy in reducing CRP (WMD −6.00, 95% CI −6.83 to −5.17, *Z* 14.09, and *P* < 0.00001, Figure 3(d)).

**Figure 4:**
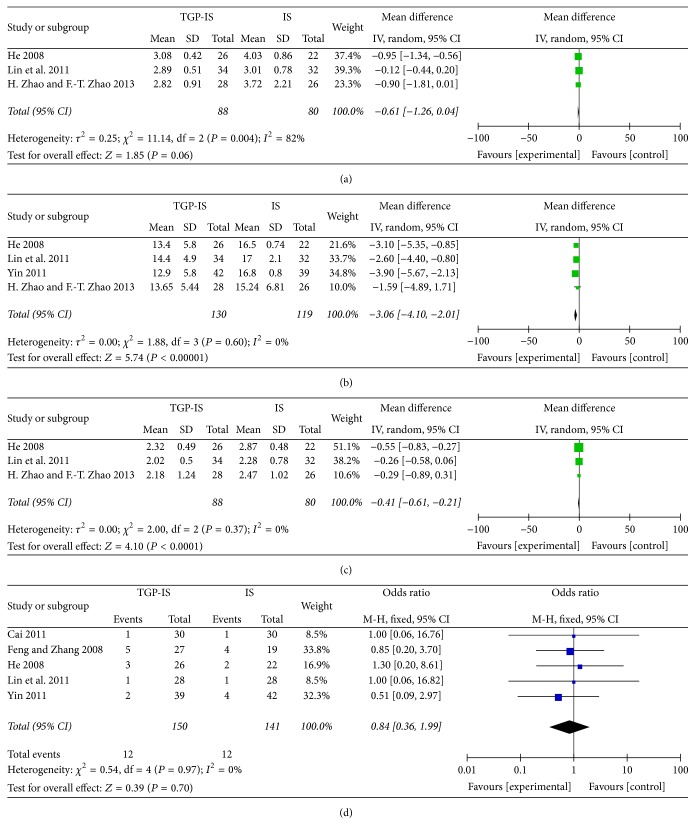
Forest plot of studies comparing TGP-IS group and the IS group, examining the effect on primary Sjögren's syndrome (including IgA, IgG, IgM, and adverse reaction). (a) IgA. (b) IgG. (c) IgM. (d) Adverse reaction.

Changes in IgA were reported in three studies [21, 24, 25]. The results revealed no obvious difference between the two groups (WMD 0.61, 95% CI −1.26 to −0.04, *Z* = 1.85, and *P* = 0.06, Figure 4(a)), with significant heterogeneity (*I*^2^ = 82%, *P* = 0.004). In addition, reductions in IgG were reported in four studies [21, 24, 25]. A fixed-effect model was used for analysis owing to the absence of statistical homogeneity among the studies (*I*^2^ = 0%, *P* = 0.60). As shown in Figure 4(b), meta-analysis indicated that TGP-IS therapy was more effective in reducing IgG than IS alone in the treatment of pSS (WMD −3.06, 95% CI −4.01 to −2.01, *Z* 5.74, and  *P* < 0.00001). Three trials reported changes in IgM [21, 24, 25], and the same trend favouring the TGP-IS combination was found in IgM analysis (WMD −0.41, 95% CI −0.61 to 0.21, *Z* 4 10, and *P* < 0.0001, Figure 4(c)).

### 3.4. Adverse Reactions

Six studies [20, 21, 23–25] reported adverse reactions. Diarrhea was a common gastrointestinal reaction in the TGP-IS group but did not require further treatment. Two studies [21, 24] reported that some patients experienced abdominal pain in the TGP-IS group. A few patients suffered vision loss, visual disturbances, skin rash, or liver function damage. In both groups, however, most adverse reactions reported were mild and gradually resolved without treatment or with symptomatic treatment. Only one patient dropped out of the experiment because of heavy diarrhea [20]. One of these studies did not report data concerning adverse reactions [25]. The adverse reaction data showed homogeneity (*P* = 0.37, *I*^2^ = 0%), and the fixed-effect model was chosen for meta-analysis. The TGP-IS group was revealed to have a lower odds ratio than that of the IS group (OR = 0.84, 95% CI 0.36 to 1.99, *Z* = 0.39 and *P* = 0.70, Figure 4(d)).

### 3.5. Sensitivity Analysis and Publication Bias

We were unable to perform sensitivity analysis due to the low methodological quality of these included trials, while no trials described the details of allocation concealment or blinding. A visual inspection of funnel plots for indicators of publication bias was not undertaken because fewer than 10 studies were analyzed [19].

## 4. Discussion

### 4.1. Efficacy of TGP for Treatment of pSS

To date, we have found only one randomized controlled trial of TGP alone in the treatment of Sjögren's syndrome. Zhou et al. [27] conducted a randomized, double-blind, placebo-controlled clinical trial in pSS patients. The results indicated that TGP appears to improve the glandular secretion function and reduce the level of inflammatory cytokines. Because of the lack of reports on TGP monotherapy for pSS, we compared TGP-IS with IS alone. The results of meta-analyses showed that TGP combined with an immunosuppressant showed greater efficacy against pSS than IS alone. The TGP-IS combination was found to be more effective in reducing inflammatory markers and immunoglobulin such as ESR, CRP, IgM, and IgG than IS alone. No significant difference was found between the two groups with regard to IgA. The TGP-IS group also improved patients' results of the saliva flow test and the Schirmer test, which were better than in the IS group. Although TGP may induce diarrhea, in these studies the diarrheal patients needed no further treatment. These results suggest that TGP are safe for the treatment of pSS. However, because of the lack of reports on TGP monotherapy for pSS and the poor quality of the included studies, we cannot fully evaluate the efficacy of TGP on the treatment of pSS.

Although the pathogenesis of pSS is not yet clear, it is generally believed that B-lymphocyte and T-lymphocyte infiltrations in the exocrine glands are characteristic of pSS [28–31]. Infiltrating lymphocytes secrete proinflammatory factors such as tumor necrosis factor-alpha, interferon-gamma, and interleukin-1 (IL-1) beta, which consequently lead to the disordered homeostasis of glandular epithelial cells and the destruction of cellular structure [32, 33]. In addition, a series of abnormal manifestations in other immune systems are involved in the pathogenetic process of pSS, such as production of autoantibodies, overexpression of Th-cells (mainly Th17), and the destruction of immune tolerance [34, 35]. Moreover, IL-17-producing CD_4_^+^CD_161_^+^T cells might be an important part of the inflammation development and B cell activation in pSS [36]. These mechanisms combined result in the occurrence and deterioration of pSS.

According to recent pharmacological research, the potential mechanism via which TGP ameliorates pSS might be associated with a multilevel regulatory mechanism. Firstly, TGP has bidirectional regulatory effects on T lymphocytes and tends to favour immune cell balance [37]. It may adjust the balance of Th1/Th2 cytokines by decreasing proinflammatory cytokines and increasing anti-inflammatory cytokines [38, 39]. In addition, TGP can inhibit maturation and activation of dendritic cells, which leads to impaired Th1/Th17 differentiation in vivo and downregulation of the Th1/Th17 inflammatory response [40, 41]. Secondly, TGP can effectively inhibit the production of B lymphocytes and improve humoral immunity [42]. Finally, TGP can significantly inhibit the progression of autoimmune inflammation, and the inhibitory effects might be associated with its ability to mediate the level of cAMP, inhibit the production of prostaglandin E, and ameliorate oxidative stress [43–45]. As a result, TGP has anti-inflammatory and immunoregulatory effects on pSS. Our research showed that TGP had no obvious toxicity, and TGP has also been reported to reduce hepatotoxicity induced by immunosuppression [15]. Therefore, TGP has a positive effect in the treatment of pSS, especially in combination with immunosuppressive agents [27].

### 4.2. Shortcomings of Current Trials

The number of clinical trials of TGP as treatment for pSS is limited. Several studies used TGP in self-controlled trials or retrospectively analyzed its efficacy compared with hydroxychloroquine [46–48]. Only one study involved a randomized double-blind placebo-controlled trial [27]. Therefore, this article focuses on the combination therapy of TGP with immunosuppressants. However, there were some deficiencies in the seven clinical studies. First, there was denormalization in the experimental design. Most studies had not reported concrete randomization and detailed allocation concealment and blinding. As a result, we could not fully judge their correctness. Secondly, there was no unified standard to evaluate the effect. Most studies adopted self-designed efficacy standards. This may hinder international exchanges and cooperation. Therefore studies of traditional Chinese medicines should try to adopt international evaluation standards to reduce bias arising from different criteria. Finally, most trials were of limited size, and all included patients were Chinese. Consequently, the research results might be limited by the influence of the geographic region and dietary habits. In short, more high-quality RCTs are needed to confirm these therapeutic effects.

## 5. Conclusion

In this review, we evaluated the clinical efficacy and safety of TGP for the treatment of pSS. The results showed that TGP combined with an immunosuppressant showed greater efficacy for improving exocrine function (saliva flow test and Schirmer test) of pSS than the same immunosuppressant alone. In addition, the same trend was found in inflammatory indices (ESR and CRP) and immunoglobulins (IgM and IgG). Adverse events were few and were similar between the two groups. So TGP appears to improve the symptoms of pSS. However, due to the low quality of included studies and the high risk of bias, further well-designed multicenter and large-scale RCTs are still needed to confirm the beneficial effects of TGP.

## Figures and Tables

**Table 1:** Characteristics of studies.

Study	EG		CG		Interventions		Course	Main outcome
	*N*	*n*	*N*	*n*	EG	CG		
Feng and Zhang 2008 [20]	42	30	36	20	TGP + MTX	MTX	3 months	A + B + D + E
He 2008 [21]	26	19	22	9	TGP + HCQ	HCQ	3 months	A + B + C + D + E
Cai 2011 [22]	30	25	30	17	TGP + MTX	MTX	6 months	A + B + C + D
Yin 2011 [23]	42	32	39	16	TGP + HCQ	HCQ	3 months	A + B + C + D
Lin et al. 2011 [24]	34	25	32	14	TGP + HCQ	HCQ	24 weeks	A + B + C + D
H. Zhao and F.-T. Zhao 2013 [25]	28	26	26	24	TGP + HCQ	HCQ	6 months	A + C + D
Liu and Deng 2016 [26]	28	24	28	16	TGP + MTX	MTX	6 months	A + B + C + D

*Note*. EG: experimental group, CG: control group, *N*: total cases, *n*: effective cases, TGP: total glucosides of paeony, HCQ: hydroxychloroquine, MTX: methotrexate, A: effective rate, B: adverse reaction, C: clinical symptoms and signs, D: laboratory indices, E: security indices.

## References

[1] Maślińska M., Przygodzka M., Kwiatkowska B., Sikorska-Siudek K. (2014). Sjögren’s syndrome: still not fully understood disease. *Rheumatology International*.

[2] Brito-Zerón P., Ramos-Casals M., E-Stf Group (2014). Advances in the understanding and treatment of systemic complications in Sjögren*ʼ*s syndrome. *Current Opinion in Rheumatology*.

[3] Zhang S.-Y. (2011). The TCM etiology, pathogenesy and differential treatment for Sjogren's syndrome. *Journal of Traditional Chinese Medicine*.

[4] Lendrem D., Mitchell S., McMeekin P. (2014). Health-related utility values of patients with primary Sjögren's syndrome and its predictors. *Annals of the Rheumatic Diseases*.

[5] Barone F., Colafrancesco S. (2016). Sjogren's syndrome: from pathogenesis to novel therapeutic targets. *Clinical and Experimental Rheumatology*.

[6] Ferro F., Vagelli R., Bruni C. (2016). One year in review 2016: Sjögren's syndrome. *Clinical and Experimental Rheumatology*.

[7] Ramos-Casals M., Tzioufas G. A., Stone H. J., Sisó A., Bosch X. (2010). Treatment of primary Sjögren syndrome: a systematic review. *Journal of the American Medical Association*.

[8] Van Nimwegen J. F., Moerman R. V., Sillevis Smitt N., Brouwer E., Bootsma H., Vissink A. (2016). Safety of treatments for primary Sjögrens syndrome. *Expert Opinion on Drug Safety*.

[9] Ma X., Chi Y.-H., Niu M. (2016). Metabolomics coupled with multivariate data and pathway analysis on potential biomarkers in cholestasis and intervention effect of Paeonia lactiflora pall.. *Frontiers in Pharmacology*.

[10] Li C. L., He J., Li Z. G., Zheng L. W., Hua H. (2013). Effects of total glucosides of paeony for delaying onset of Sjogren's syndrome: an animal study. *Journal of Cranio-Maxillofacial Surgery*.

[11] Luo J., Jin D.-E., Yang G.-Y. (2016). Total glucosides of paeony for rheumatoid arthritis: A protocol for a systematic review. *BMJ Open*.

[12] Zhang H.-F., Xiao W.-G., Hou P. (2011). Clinical study of total glucosides of paeony in patients with systemic lupus erythematosus. *Chinese Journal of Integrated Traditional and Western*.

[13] Zhang H.-F., Hou P., Xiao W.-G. (2007). Clinical observation on effect of total glucosides of paeony in treating patients with non-systemic involved Sjogren syndrome. *Chinese Journal of Integrated Traditional and Western*.

[14] Qin Y., Tian Y.-P. (2011). Protective effects of total glucosides of paeony and the underlying mechanisms in carbon tetrachloride-induced experimental liver injury. *Archives of Medical Science*.

[15] Chen Z., Li X.-P., Li Z.-J., Xu L., Li X.-M. (2013). Reduced hepatotoxicity by total glucosides of paeony in combination treatment with leflunomide and methotrexate for patients with active rheumatoid arthritis. *International Immunopharmacology*.

[16] Xiang N., Li X.-M., Zhang M.-J. (2015). Total glucosides of paeony can reduce the hepatotoxicity caused by Methotrexate and Leflunomide combination treatment of active rheumatoid arthritis. *International Immunopharmacology*.

[17] Wang Y.-W., Wang Y.-J. (2007). Pharmacological study and clinical application of total glucosides of peony in autoimmune diseases. *Journal of Zhejiang University of Chinese Medicine*.

[18] Vitali C., Bombardieri S., Jonsson R. (2002). Classification criteria for Sjögren's syndrome: a revised version of the European criteria proposed by the American-European consensus group. *Annals of the Rheumatic Diseases*.

[19] Cochrane Handbook for Systematic Reviews of Interventions Ver-sion 5. 1. 0. http://www.cochrane-handbook.org.

[20] Feng F.-H., Zhang E.-Z. (2008). 42 cases of Sjögren's syndrom treated by total glucosides of peony. *Traditional Chinese Medicinal Research*.

[21] He H. (2008). Clinical investigation of Sjögren's syndrome treated with total glucosides of peony alliance with hydroxychloroquine. *Hubei Journal of Traditional Chinese Medicine*.

[22] Cai W.-H. (2011). Clinical investigation of Sjögren's syndrome treated with total glucosides of peony. *Contemporary Medicine*.

[23] Yin B. (2011). Clinical investigation of Sjögren's syndrome treated with total glucosides of peony alliance with hydroxychloroquine. *World Chinese Journal of Digestology*.

[24] Lin X.-Y., Chen R.-L., Zhu L.-X. (2011). Clinical observation on effect of total glucosides of paeony in treatment of primary Sjögren's syndrome. *Journal of Aesthetics and Cosmetology*.

[25] Zhao H., Zhao F.-T. (2013). Clinical study on treatment of primary Sjögren's syndrome by total glucosides of paeony and hydroxychloroquine. *Henan Traditional Chinese Medicine*.

[26] Liu Y., Deng H. (2016). Clinical investigation of Sjögren's syndrome treated by total glucosides of peony alliance with methotrexate. *Chinese Journal of General Practice*.

[27] Zhou Y., Jin L., Kong F. (2016). Clinical and immunological consequences of total glucosides of paeony treatment in Sjögren's syndrome: a randomized controlled pilot trial. *International Immunopharmacology*.

[28] Christodoulou M. I., Kapsogeorgou E. K., Moutsopoulos H. M. (2010). Characteristics of the minor salivary gland infiltrates in Sjögren's syndrome. *Journal of Autoimmunity*.

[29] Nocturne G., Mariette X. (2013). Advances in understanding the pathogenesis of primary Sjögren's syndrome. *Nature Reviews Rheumatology*.

[30] Alunno A., Carubbi F., Bistoni O. (2014). CD4-CD8- T-cells in primary Sjögren's syndrome: association with the extent of glandular involvement. *Journal of Autoimmunity*.

[31] Jonsson R., Vogelsang P., Volchenkov R., Espinosa A., Wahren-Herlenius M., Appel S. (2011). The complexity of Sjögren's syndrome: Novel aspects on pathogenesis. *Immunology Letters*.

[32] Barrera M. J., Bahamondes V., Sepúlveda D. (2013). Sjögren's syndrome and the epithelial target: A comprehensive review. *Journal of Autoimmunity*.

[33] Baker O. J., Camden J. M., Redman R. S. (2008). Proinflammatory cytokines tumor necrosis factor-*α* and interferon-*γ* alter tight junction structure and function in the rat parotid gland Par-C10 cell line. *American Journal of Physiology - Cell Physiology*.

[34] Alunno A., Bistoni O., Bartoloni E. (2013). IL-17-producing CD4-CD8- T cells are expanded in the peripheral blood, infiltrate salivary glands and are resistant to corticosteroids in patients with primary Sjögren's syndrome. *Annals of the Rheumatic Diseases*.

[35] Abdulahad W. H., Kroese F. G. M., Vissink A., Bootsma H. (2012). Immune regulation and B-cell depletion therapy in patients with primary Sjögren's syndrome. *Journal of Autoimmunity*.

[36] Li L., He J., Zhu L. (2015). The clinical relevance of IL-17-producing CD4+CD161+ cell and its subpopulations in primary sjögren's syndrome. *Journal of Immunology Research*.

[37] Wu G., Wu N., Li T., Lu W., Yu G. (2016). Total glucosides of peony ameliorates sjögren's syndrome by affecting TH1/TH2 cytokine balance. *Experimental and Therapeutic Medicine*.

[38] Wang C., Yuan J., Wu H.-X. (2015). Total glucosides of paeony inhibit the inflammatory responses of mice with allergic contact dermatitis by restoring the balanced secretion of pro-/anti-inflammatory cytokines. *International Immunopharmacology*.

[39] Zhang Y., Zhou R., Zhou F., Cheng H., Xia B. (2014). Total Glucosides of Peony Attenuates 2,4,6-Trinitrobenzene Sulfonic Acid/Ethanol-Induced Colitis in Rats Through Adjustment of Th1/Th2 Cytokines Polarization. *Cell Biochemistry and Biophysics*.

[40] Lin J., Xiao L., Ouyang G. (2012). Total glucosides of paeony inhibits Th1/Th17 cells via decreasing dendritic cells activation in rheumatoid arthritis. *Cellular Immunology*.

[41] Zhou Z., Lin J., Huo R. (2012). Total glucosides of paeony attenuated functional maturation of dendritic cells via blocking TLR4/5 signaling in vivo. *International Immunopharmacology*.

[42] Song S. S., Yuan P. F., Li P. P. (2015). Protective Effects of Total Glucosides of Paeony on N-nitrosodiethylamine-induced Hepatocellular Carcinoma in Rats via Down-regulation of Regulatory B Cells. *Immunological Investigations*.

[43] Wang Y. N., Zhang Y., Wang Y. (2014). The beneficial effect of total glucosides of paeony on psoriatic arthritis links to circulating tregs and th1 cell function. *Phytotherapy Research*.

[44] Wang Y., Zhang H., Du G. (2016). Total glucosides of paeony (TGP) inhibits the production of inflammatory cytokines in oral lichen planus by suppressing the NF-*κ*B signaling pathway. *International Immunopharmacology*.

[45] Su J., Zhang P., Zhang J.-J., Qi X.-M., Wu Y.-G., Shen J.-J. (2010). Effects of total glucosides of paeony on oxidative stress in the kidney from diabetic rats. *Phytomedicine*.

[46] Feng L., Ma L. (2013). Clinical efficacy of total glucosides of paeony in the treatment of primary Sjögren's syndrome. *West China Medical Journal*.

[47] Lu J., Shen H., Liu Y. (2006). Clinical observation of total glucosides of paeony on Sjögren's syndrome. *China Journal of Modern Medicine*.

[48] Wang H. (2008). Clinical observation of total glucosides of paeony on Sjögren's syndrome. *Gan Su Journal of Traditional Chinese Medicine*.

